# Category-Based Learning About Deviant Outgroup Members Hinders Performance in Trust Decision Making

**DOI:** 10.3389/fpsyg.2018.01008

**Published:** 2018-06-21

**Authors:** Maïka Telga, Soledad de Lemus, Elena Cañadas, Rosa Rodríguez-Bailón, Juan Lupiáñez

**Affiliations:** ^1^Cognitive Neuroscience Lab, Centro de Investigación Mente, Cerebro y Comportamiento, Department of Experimental Psychology, Universidad de Granada, Granada, Spain; ^2^Social Psychology Lab, Centro de Investigación Mente, Cerebro y Comportamiento, Department of Social Psychology, Universidad de Granada, Granada, Spain; ^3^Akili Interactive Labs, Boston, MA, United States

**Keywords:** categorization, individuation, motivation, trust, outgroup homogeneity

## Abstract

The present research examines whether individuation and categorization processes influence trust decisions about strangers at first and across repeated interactions. In a partial replication of the study reported by Cañadas et al. (2015), participants played an adaptation of the multi-round trust game paradigm and had to decide whether or not to cooperate with unknown partners. Gender (Study 1a) and ethnicity (Studies 1b, 2, and 3) served to create distinct social categories among the game partners, whose reciprocation rates were manipulated at group and individual levels. At the group level, two social groups (i.e., ingroup vs. outgroup) were associated with opposite reciprocation rates (i.e., high vs. low reciprocation rate). At the individual level, consistency was manipulated by altering the reciprocation rate of one out of four members of each social group. That is, there was one inconsistent individual in each group showing a pattern of reciprocation opposite to the group reciprocation rate. Our data, contrary to Cañadas et al.’s (2015) findings, suggested that ingroup partners were individuated given that participants made their decisions to cooperate with the trustees according to their individual reciprocation rate and independently of the group reciprocation rate. In contrast, decisions about outgroup partners (i.e., men in Study 1a and Blacks in Studies 1b, 2, and 3) were affected by category-based thinking. At the same time, in comparison with ingroup, greater cooperation was observed with ethnic outgroups but not with gender outgroups. The consistency of our results with the previous literature on social categorization and across the three experiments seems to indicate they are reliable, supporting the hypothesis that categorization and individuation processes guide trust decision-making, promoting individuation mainly for ingroup and categorization among outgroup members.

## Introduction

In our daily life, plenty of situations require us to make decisions about people we do not know, from helping a beggar to hiring someone’s services. When we get involved in these interactions, we surely have a confident positive expectation regarding the behavior of these people, that is, we trust them (Lewicki et al., 1998). But, once we decide to interact with them, we have to deal with uncertainty since we have no further control on the outcomes. This is why trust has often been considered as irrational or inconsistent with self-interested decisions (Berg et al., 1995). Indeed, trusting someone unknown is risky given one exposes him/herself to deception or exploitation. In fact, trust has also been defined as an “intention to accept vulnerability” (Dunning et al., 2014, p. 123). But it is also “an important lubricant of social system” (Arrow, 1974, p. 23) since trust promotes cooperation between individuals (Barnard, 1968; Deutsch, 1973), which in turn leads to reciprocity in addition to being rewarding on its own (Tomasello, 2009). Most theories consider that people engage in trust behaviors when their tolerance of risk has not been trespassed (Dunning et al., 2014). However, often people trust strangers with whom they have no prior experience thus bearing a very high risk of deception (Johnson and Mislin, 2011; Wilson and Eckel, 2011). This paradox has been investigated in psychology (e.g., Balliet and Van Lange, 2013), sociology (e.g., Paxton, 2001) or political science (e.g., Wilson and Eckel, 2011). But the topic particularly caught attention among economists who have provided theories and procedures to examine how we engage in interactions involving trust (Johnson and Mislin, 2011).

The trust game (Berg et al., 1995) is a useful paradigm to investigate under which circumstances people place their trust in someone else’s hands. In its classical version, participants are endowed with 10$ and have to decide how much of this initial amount they will send to an anonymous partner. In a second stage, the amount sent is tripled and participants’ partner can decide how much of the received money, if any, they would send back to the participant. Thus, participants are “trustors,” whereas the partner is the “trustee” who has the power to make a decision that affects both the trustors and themselves. From the participants’ perspective, the most rational decision is to send nothing since they have no guarantee to receive something back. But research has shown that participants do trust strangers who, in turn, reciprocate (Johnson and Mislin, 2011; Wilson and Eckel, 2011; Balliet and Van Lange, 2013). Therefore, rational decision-making based on risk attitudes is not sufficient to explain how we decide to place our trust in someone. Indeed, several social factors such as socioeconomic status (e.g., Blue et al., 2018; Bogliacino et al., 2018), emotion (e.g., Tortosa et al., 2013b; Alguacil et al., 2017), or face appearance (e.g., van ’t Wout and Sanfey, 2008; Li et al., 2017) have been shown to affect trust decision-making at zero acquaintance. All the social variables (e.g., facial expression, gaze direction, gender, ethnicity, attractiveness) that might influence the impression formation process (Uleman and Kressel, 2013; Stolier and Freeman, 2016) can in turn affect the decisions being made. Understanding processes underlying trust decisions requires understanding what factors influence social perception and impression formation.

Social stimuli are complex and contain considerable information. Body language (e.g., Tiedens and Fragale, 2003; de Lemus et al., 2012), facial expression (e.g., Cañadas et al., 2016), gaze direction (e.g., Macrae et al., 2002), skin color (e.g., Sommers, 2006), gender and attractiveness (Solnick and Schweitzer, 1999) are some of the numerous cues which influence our perception and expectations about strangers. Processing social information is cognitively demanding so we need to deal with this information efficiently. Social categorization allows us to make sense of our social world effortlessly (e.g., Fiske and Neuberg, 1990) by using noticeable information to classify others on the basis of the diagnostic characteristics of the social groups to which they belong. Categorization is a prominent strategy when we perceive social stimuli (Brewer, 1988; Fiske and Neuberg, 1990; Fiske et al., 1999; Macrae and Bodenhausen, 2000; Cuddy et al., 2004), but this basic tendency to attend to social information can be overcome by activating instead the motivation to focus on individuating characteristics. Indeed, several factors such as prejudice level (Lepore and Brown, 1997), personal relevance (Fiske and Neuberg, 1990), instructions (Cañadas et al., 2013), power (Goodwin et al., 2000; Rodríguez-Bailón et al., 2000), interdependence (Bukowski et al., 2009) or some contextual variables (Blair, 2002) can selectively direct attention toward individual-based features, thus allowing to discriminate among individuals within a social group. Given the potential negative consequences of misattribution of traits, being able to flexibly adopt individuation or categorization based strategies is crucial for understanding our social world.

Once social categories are established, one necessarily realizes that he/she falls into some social groups (i.e., ingroups), and remains excluded from others (i.e., outgroups) (Ellemers and Haslam, 2012). These processes of self-categorization are crucial for self-perception. Depending on the context, people can categorize themselves according to different social identities (e.g., gender, ethnicity, etc.) which are associated with different emotional significance (Turner et al., 1987; Ellemers and Haslam, 2012). The saliency of the social identity and the relevance of the ingroup for the self will determine how much are people willing to use certain strategies to enhance the group identity (Tajfel, 1978), broadly resulting in a more positive attitude toward ingroup than outgroup members (Tajfel and Turner, 1979). Such ingroup bias can be observed in a large range of responses, from resources distribution (Tajfel et al., 1971) to empathy (Xu et al., 2009), including trust (Wilson and Kayatani, 1968; Tanis and Postmes, 2005; Romano et al., 2017). Thus, the motivation to enhance or maintain a positive social identity should lead people to cooperate more with ingroup than with outgroup members (Brewer, 2008).

Beyond group identity, cooperation can lead to more global positive outcomes such as humans’ survival. From an evolutionary approach, group organization allows to establish an exchange network necessary for survival (Henrich and Henrich, 2007). According to the Bounded Generalized Reciprocity theory, cooperative individuals within a group help to achieve this goal and gain the reputation of being reliable cooperators, which enhances their probability to remain part of the group (Yamagishi et al., 1999). Importantly, when it comes to trust decision-making in intergroup contexts, both interests in achieving a positive social identity or maximizing the groups’ outcomes converge in promoting ingroup favoritism and intergroup discrimination.

As well as ingroup bias, a different consequence of social categorization is reflected in the outgroup homogeneity effect (Tajfel and Wilkes, 1963), that is, a category-based perception of the outgroup resulting in a greater perceived similarity among outgroup members than among ingroup members, for both physical features directly observable (see Meissner and Brigham, 2001; Hugenberg et al., 2010 for a review) and more complex personality traits (Linville et al., 1986, 1989; Freeman et al., 2010). For example, same-race faces are better recognized (Hugenberg et al., 2010) and differentially attended (Kawakami et al., 2014) than other-race faces. Therefore, the use of social categories to extract information about unknown people has important consequences for our judgment, our expectations from others, and in general the way we interact with them (Allport, 1954; Macrae and Bodenhausen, 2000; Bodenhausen et al., 2012; Kawakami et al., 2017). Altogether, these effects suggest that despite their cognitive efficiency, social categorization processes might also lead us to biased perception and flawed decision-making. For instance, the outgroup homogeneity effect can lead to overgeneralization, failure to distinguish among the members of the same category and stereotyping (Allport, 1954; Levin, 1996, 2000; Stroessner, 1996; Blair et al., 2004). In cooperation settings, individuation should be a more efficient strategy, leading to more accurate predictions of people’s cooperative tendency. In this research, we aim to deepen our understanding of how categorization and individuation processes are used in social interactions, and how they modulate the way we learn who is trustworthy.

In an attempt to clarify whether categorization and individuation processes affect the way we learn whether to trust unknown game partners depending on their ethnicity, Cañadas et al. (2015) conducted an adaptation of the trust game paradigm. They used the multi-round version of the trust game (King-Casas et al., 2005) in which participants interact several times with the trustees. Because of these repeated interactions, participants’ best strategy is to individuate and learn as fast as possible the reciprocation rate of each trustee. In Cañadas et al.’s (2015) adaptation, all participants were white and played with white and black trustees. Each ethnic group was associated with either a high or a low proportion of reciprocation rate. For instance, black trustees reciprocated in 75% of the trials whereas white trustees reciprocated only in 25% of the trials. Furthermore, in each group, one individual was inconsistent with respect to the other members, that is, this person was associated with the reciprocation rate corresponding to the other ethnic group. Following the same example, one black partner tended not to reciprocate whereas one white partner highly reciprocated. With this procedure, participants’ cooperation strategies toward the inconsistent individual are critical. If participants individuate their partners, they should cooperate with the inconsistent individual according to his or her own reciprocation rate and independently of the group reciprocation rate. On the other hand, if participants categorized their partners, they should apply the group reciprocation knowledge to the inconsistent individual.

Participants were expected to mostly individuate their game partners using the trial-by-trial feedback to guide their decisions, given this strategy is the one that maximizes profits. Moreover, and according to previous research, this pattern was expected mainly for ingroup members who are generally perceived along with an identity-based diagnostic rather than categorization processes (Tajfel and Wilkes, 1963). Contrary to this hypothesis, participants showed a pattern of categorization for ingroup members (white trustees) and a pattern of individuation for outgroup members (black trustees). Cañadas et al. (2015) argued that despite the manipulation of reciprocation rates according to trustees’ ethnicity, a different social dimension may have been salient and confused participants as men and women were included within each ethnic group. Therefore, participants may have relied on gender over ethnicity to identify and categorize their game partners, so that ethnicity did not have the relevance expected in their experiment. In the current research, we go beyond the previous study reported by Cañadas et al. (2015) by experimentally distinguishing the effects of race and gender on trust decision-making. We adapted and replicated Cañadas et al.’s (2015) procedure across three experiments in which we also investigated a possible effect of experimenter’s ethnicity on participants’ decisions.

One of the main goals of the present research was to clarify the results reported by Cañadas et al. (2015) by disentangling the effects of gender and ethnicity in a multi-round trust game task. In order to achieve this goal, we manipulated between experiments trustees’ group membership for participants to use just one social dimension, while the others remained constant across all trustees. That is, we presented participants with men and women, all belonging to the participants’ ethnic group (white; Study 1a), or with blacks and whites, all belonging to the participants’ same gender category (women; Study 1b). In Study 2, we focused on the ethnic category and repeated the same experimental procedure as in Study 1b introducing a between-group manipulation of the experimenter’s ethnicity. This allowed us to explore a possible effect of social desirability boosted by the presence of an outgroup experimenter, which may have influenced participants’ responses. Finally, in Study 3, we directly replicated the experiment reported by Cañadas et al. (2015) including men and women in each ethnic group, while we maintained the between-group manipulation of experimenter’s ethnicity.

In line with Cañadas et al.’s (2015) general prediction, we expected participants to mostly individuate their partners since they were provided with both the motivation (i.e., economic outcomes) and the means (i.e., feedback after each trial) to do so. Moreover, since the saliency of gender may have confounded the identification of ethnicity as the relevant social dimension in Cañadas et al.’s (2015) study, we had no strong theoretical motives to expect a replication of the data they reported. Thus, in Studies 1 and 2, we predicted on both gender and ethnicity dimensions a stronger pattern of individuation for ingroup members than for outgroup members as suggested by the previous literature (Tajfel and Wilkes, 1963), in contrast to the pattern observed by Cañadas et al. (2015). Finally, in Study 3, we directly replicated the experimental design of Cañadas et al. (2015) while testing for a possible experimenter effect.

## Study 1

The goal of Study 1 was to distinguish between the effects of gender and ethnicity in a trust game paradigm. In order to achieve this, participants (all white female) were randomly assigned to one of two experiments: in Study 1a gender was manipulated while ethnicity remained constant across all trustees (all white) whereas in Study 1b ethnicity was manipulated while gender was identical across all trustees (all women).

### Method

#### Participants

Studies 1a and 1b were conducted concurrently and participants were randomly assigned to one of the two conditions^1^. Forty^2^ Caucasian female students (mean age: 20.48 years, range: 18–26 years) participated in Study 1a and 41 Caucasian female students (mean age: 20.11 years, range: 18–27 years) participated in Study 1b. All the participants were volunteers from the local university who took part in exchange for financial compensation according to their performance in the task (5.82€ on average). In these experiments and the following ones, all participants had normal or corrected to normal vision and were naïve as to the purpose of the study. Written informed consent was obtained from all participants and the experiments were conducted according to the guidelines set forth by the local university on the use of human participants in research.

#### Apparatus and Stimuli

PCs with E-Prime 2.0 software package (Schneider et al., 2002) were used for stimuli presentation and data acquisition. Stimuli were presented on a 17-in. computer screen and consisted of full color photographs of an emotionally neutral face with a direct gaze on a gray background. The photographs were taken from the NimStim Set of Spatial Expressions (Tottenham et al., 2009) as Cañadas et al. (2015). However, given that we needed to introduce more faces of white (Study 1) or black trustees (Study 1b), some of the stimuli were taken from a different database^3^. Overall, 24 different photographs were used to represent the trustees, corresponding to eight white women (Studies 1a and 1b), eight white men (Study 1a), and eight black women (Study 1b).

#### Procedure

As a cover story, the experimenter explained to participants that they would take part in a study about economic decision-making. Participants were motivated to be as accurate as possible as they would be economically rewarded proportionally to their performance in the task.

Participants played a multi-round trust game adapted from King-Casas et al. (2005). Each trial consisted of a game with a virtual partner represented by one of the 24 faces that served as stimuli. At the beginning of the trial, participants were presented for 190 ms with a euro symbol (i.e., “€”) indicating the endowment of 1€. Then, a fixation cross was presented during 500 ms followed by the photograph of the game partner for 1,500 ms. Participants were asked to indicate whether or not they decided to cooperate with the corresponding trustee by pressing the ‘1’ key in case of cooperating and the ‘0’ key in the contrary case. In case of cooperating, their game partner would receive the initial 1€ multiplied by 5 (i.e., 5€) with the possibility to either keep the whole money for their own, or to reciprocate by sending back half of the amount (i.e., 2.5€). In case of not cooperating, participants would keep the initial 1€ for themselves and their partner would receive nothing. Once they responded (or after 1,500 ms), a second fixation cross was presented for 500 ms followed by visual feedback displayed for 1,000 ms. In order to display feedback, we used three symbols (i.e., “o,” “∗,” and “#”) in three different colors (i.e., blue, brown, and green). Each of them was associated with the following meanings: “You have kept the money,” “You have cooperated and your partner has reciprocated,” and “You have cooperated and your partner has not reciprocated.” The association between symbols, colors and their meanings was counterbalanced across participants. The message “You did not answer” was displayed when participants did not answer after 1,500 ms. Feedback was displayed after each trial all along the task.

**FIGURE 1:**
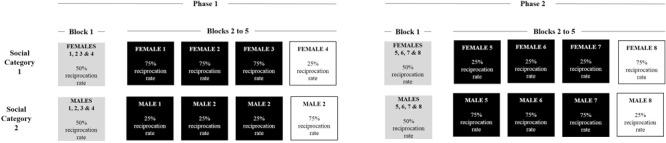
Example of reciprocation rates in Study 1a, adapted from Cañadas et al. (2013).

We manipulated trustees reciprocation on the basis of the procedure developed by Cañadas et al. (2015), as seen in **Figure 1**. Concretely, trustees’ reciprocation was manipulated at the group and the individual level. At the group level, the aforementioned ingroup-outgroup distinctions (Study 1a: women vs. men; Study 1b: blacks vs. whites) were used to create an association between social categories and a particular reciprocation rate. Ingroup and outgroup displayed opposite patterns of reciprocation, that is, when one group (e.g., the ingroup) was associated with a high proportion of reciprocation (i.e., reciprocating in 75% of the trials), the other one (e.g., outgroup) was associated with a low reciprocation rate (i.e., reciprocating in 25% of the trials). At the same time, reciprocation rate was manipulated at the individual level: consistent individuals (three out of four members) displayed the same reciprocation rate as the category they belonged to, whereas inconsistent individuals (one out of four members) were associated with the opposite pattern of reciprocal cooperation. Faces associated with consistent or inconsistent conditions were counterbalanced across participants.

Altogether, participants played 40 times with each of the 16 trustees, resulting in 640 trials. The task was divided in two phases of five blocks each (**Figure 1**). In the first phase, in Block 1, all trustees reciprocated at 50%. This block was introduced in order to verify whether participants presented a prior bias in their decision-making, which would be reflected in a higher cooperation rate with one of the two groups. Further, in Blocks 2–5, we implemented the cooperation manipulations, i.e., group reciprocation and individual consistency, by associating members of each group with a particular reciprocation rate, as described above. In the second phase, in Block 1 we introduced eight new faces (four from each social category) with which participants had no prior experience and belonging to the two social categories used. Again, trustees started cooperating at 50%, which allowed us to analyze whether the learning from the first phase generalized to new individuals in the second phase. This would be reflected in a biased cooperation rate toward one group depending on which group started reciprocating in a higher or lower proportion of trials during the first phase. Finally, in Blocks 2–5 of the second phase, we reversed the association between the groups and their reciprocation rate. For instance, if the members of the ingroup “women” were associated with a high reciprocation rate in the first phase, the new faces of women were later associated with a low reciprocation rate in the second phase. The order in which ingroup or outgroup started with a high reciprocation rate was counterbalanced across participants. This procedure was used in order to have a full within-participants design regarding the variables of interest.

At the end of the experiment, participants were endowed along with their percentage of accuracy^4^ in the task.

#### Design

In this study and the following ones, we manipulated four within-participants independent variables (IVs) corresponding to trustees’ social group membership (i.e., trustee group: ingroup vs. outgroup), trustees’ group reciprocation trend (i.e., group reciprocation: high vs. low), trustees’ consistency with respect to the reciprocation trend of the other members of their group (i.e., individual consistency: consistent vs. inconsistent), and the block of trials numbered from the first presentation of unknown faces (i.e., blocks: 1–5 from both phases). The dependent variable (DV) was participants’ cooperation rate.

### Results

Three participants (all from Study 1b) were excluded from the analyses for having a mean RT shorter than 200 ms in more than 50% of the trials (which was considered as a signal that participants did not thoroughly respond to faces). Furthermore, we applied the same criterion as Tortosa et al. (2013a) and excluded trials with RTs shorter than 200 ms from the analyses (4% in Study 1a and 5 % in Study 1b).

First, the cooperation rates for Blocks 1 of each phase (in which participants were exposed to unknown faces) will be presented, followed by the analyses of cooperation rates across Blocks 2–5 of both phases, from which it can be deduced whether participants learned about trustees as individuals or as members of their group.

#### Cooperation Rates Prior to Learning: Block 1 of Each Phase

In order to verify whether participants’ decision to cooperate was biased toward one or the other social group, we analyzed their cooperation rate in **Block 1** of the first phase (in which reciprocation rate was at 50%) as a function of the trustees’ group membership (i.e., trustee group). Thus, we conducted a repeated-measures Analysis of Variance (ANOVA) on cooperation rates with trustee group as a within-participants factor. In Study 1a, when the outgroup was manipulated on gender dimension, we observed a significant effect of the trustee group factor, *F*(1,39) = 4.63, *p* = 0.04, $\eta_{p}^{2}$ = 0.11, showing that participants cooperated more with women (the ingroup, *M* = 0.68, *SD* = 0.16, CI: 0.63–0.73) than with men (the outgroup, *M* = 0.61, *SD* = 0.19, CI: 0.54–0.66), thus showing an ingroup favoritism. In Study 1b, in which participants played with black and white female trustees, we also observed a significant effect of the trustee group factor, *F*(1,37) = 7.58, *p* = 0.01, $\eta_{p}^{2}$ = 0.17. However, the results indicated that participants cooperated more with black trustees (the outgroup, *M* = 0.70, *SD* = 0.18, CI: 0.64–0.76) than with white ones (the ingroup, *M* = 0.60, *SD* = 0.22, CI: 0.53–0.67), thus showing an outgroup favoritism.

On the other hand, in order to verify whether the experience with trustees in the first part of the experiment (Blocks 2–5 of the first phase) had influenced the interaction with new individuals from the same social categories, we analyzed participants’ cooperation rates in **Block 1** of the second phase depending on the trustees’ group membership (i.e., trustee group) and more critically, on which group highly cooperated in Blocks 2–5 of the first phase (i.e., first cooperator). We conducted a mixed-design ANOVA with trustee group (ingroup vs. outgroup) as a within-participant variable and first cooperator (ingroup vs. outgroup) as a between-group variable on cooperation rate in Block 1 of the second phase. We observed no significant effects, all *F*s < 1.4, *p*s > 0.24.

#### Cooperation Rates as a Result of Learning: Blocks 2–5 of Both Phases

##### Study 1a. (White) women vs. men trustees

In order to verify how gender categorization or individuation strategies had been used within this task, we analyzed participants’ cooperation rates with trustees according to their group membership (i.e., trustee group), the reciprocation trend displayed by the group (i.e., group reciprocation), the consistency of each trustee respecting the other members of their group (i.e., individual consistency) and the block of trials (i.e., blocks). If participants categorized, this should be reflected in a main effect of group reciprocation (and the absence of Group Reciprocation × Individual Consistency interaction), such that participants cooperate in the same way with both consistent and inconsistent individuals within a social category. In contrast, if participants individuated, the effect of group reciprocation should interact with the individual consistency variable, that is, participants should reverse their reciprocation rates with inconsistent trustees. Therefore, we conducted a repeated-measures ANOVA on cooperation rate with trustee group (ingroup vs. outgroup), group reciprocation (high vs. low), individual consistency (consistent vs. inconsistent) and blocks (2–5) as within-participants factors.

**FIGURE 2:**
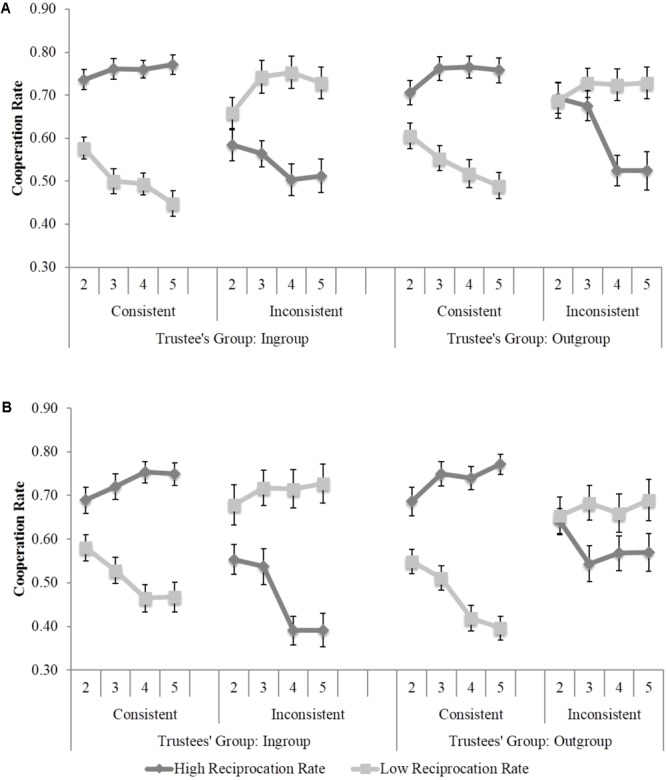
Participants’ cooperation rates in Study 1 as a function of trustees’ group, individual consistency and blocks of trials. In this figure and the following ones, Blocks 2–5 referred to the learning blocks in both phases of the experiment and error bars represent the standard error of the mean retrieving the within-participants variability as in Cousineau (2005). **(A)** Study 1a: Gender. **(B)** Study 1b: Ethnicity.

We found a significant Group Reciprocation × Individual Consistency interaction, *F*(1,39) = 42.31, *p* < 0.001, $\eta_{p}^{2}$ = 0.52 indicating that participants’ decision to cooperate was not solely led by trustees’ group but also by individual features. Indeed, the effect of individual consistency (an opposite pattern of cooperation rates for inconsistent and consistent individuals) indicated that within each group, participants identified inconsistent individuals and efficiently adjusted their cooperation rates with them. Moreover, this interaction was moderated by blocks, *F*(3,117) = 18.67, *p* < 0.001, $\eta_{p}^{2}$ = 0.32, suggesting that this pattern of individuation emerged all along the task as a result of learning, as shown in **Figure 2**. We also found a significant Trustee Group × Group Reciprocation × Individual Consistency interaction, *F*(1,39) = 4.43, *p* = 0.04, $\eta_{p}^{2}$ = 0.10, suggesting that despite participants learnt to individuate all along the task, for both ingroup, *F*(1,39) = 54.59, *p* < 0.001, $\eta_{p}^{2}$ = 0.58, and outgroup members, *F*(1,39) = 21.54, *p* < 0.001, $\eta_{p}^{2}$ = 0.37, the pattern of individuation was stronger for the ingroup, as shown in **Figure 2**.

##### Study 1b. female black vs. white trustees

The same analysis was conducted in Study 1b, in which ethnicity served to distinguish between ingroup (white) and outgroup (black) members. Again, we found a significant Group Reciprocation × Individual Consistency × Blocks interaction, *F*(3,111) = 19.27, *p* < 0.001, $\eta_{p}^{2}$ = 0.34, showing that participants mainly individuated their game partners and this tendency increased all along the blocks of trials. The Trustee Group × Group Reciprocation × Individual Consistency interaction was marginal, *F*(1,37) = 2.97, *p* = 0.09, $\eta_{p}^{2}$ = 0.07), reflecting a pattern of individuation for both ingroup, *F*(1,7) = 43.35, *p* < 0.001, $\eta_{p}^{2}$ = 0.54, and outgroup members, *F*(1,37) = 34.78, *p* < 0.001, $\eta_{p}^{2}$ = 0.49, although also in this case the pattern of individuation tended to be stronger for the ingroup (**Figure 2**).

### Discussion

Study 1 aimed at exploring the effects of gender and ethnicity on participants’ decisions to trust unknown game partners in a multi-round trust game task. Prior to experience, we expected participants to identify and use social categories to guide their decisions, such that they would trust more ingroup (i.e., women in Study 1a and Whites in Study 1b) than outgroup members (i.e., men in Study 1a and Blacks in Study 1b). Our data were consistent with this hypothesis in Study 1a since our sample of women cooperated more with women than with men. Nonetheless, we found the opposite pattern in Study 1b, when the ingroup-outgroup distinction was manipulated based on ethnicity. That is, our white participants cooperated more with black than with white partners.

While inquiring ourselves about this pattern of results we noticed that the experimenter in this study was a black woman, something very unusual in the context in which the experiment took place. Therefore, we thought that this pattern might be a result of the *experimenter effect* (Sattler, 1970) because of the presence of a black woman experimenter in both studies. Lowery et al. (2001) argued that the presence of a black experimenter might be a tacit form of social influence that increases participants’ social regulation. According to them, social regulation is determined by presumptions about the attitude of others and relationship-specific motives. In Study 1a, the experimenter was an ingroup member according to the salient social dimension manipulated in the task (i.e., gender). In contrast, in Study 1b, the experimenter was an outgroup member considering the relevant dimension for performing the task (i.e., ethnicity). Therefore, participants might have been more likely concerned about possible discrepancies between their own attitudes toward black people and the experimenter’s ones in Study 1b, than in Study 1a. This may have led them to be particularly careful not to be perceived as holding prejudices against black people such that participants’ cooperation rates with black trustees in Block 1 of the first phase may have been artificially enhanced because of the presence of a black experimenter. This issue is addressed in Studies 2 and 3.

Moreover, given that participants played several times with the same partners, the intrinsic motivation reinforced by economic outcomes, and the feedback after each trial, we expected participants to adjust their strategies all along the task in Blocks 2–5. Particularly, we argued that participants would use differentially categorization and individuation processes to guide their decision according to trustees’ group membership. We expected ingroup members (e.g., white women in both experiments) to be individuated, that is, participants would use their personal characteristics in order to decide whether or not to trust them. However, given that outgroup members are perceived more categorically (i.e., outgroup homogeneity effect, Tajfel and Wilkes, 1963) we expected this pattern to be weaker for outgroup members. The data supported our hypotheses in both Studies 1a and 1b. While participants made their decision of cooperation according to the individual reciprocation trend of each trustee, cooperation decisions with male (Study 1a) and black (Study 1b) trustees were somehow influenced by the group reciprocation trend. Specifically, participants were less efficient at making decisions about outgroup members when they did not reciprocate accordingly to the rest of their group (i.e., inconsistent individuals), as shown in **Figure 2**, despite the 40 interactions with each trustee. This suggests that when making decisions about the outgroup, the group reciprocation trend was particularly relevant, reflecting a more category-based decision for outgroup than ingroup members on gender dimension. This effect was only marginally significant in Study 1b, in which trustees’ ethnicity was manipulated. It is possible that the experimenter effect described above affected not only participants’ spontaneous cooperation attitudes with black trustees (Block 1 of the first phase), but also their learning about black trustees’ cooperation trends (Blocks 2–5 of both phases). As a consequence of an increased social regulation, participants may have paid more attention to black trustees, and therefore showed a better performance at the task. Interestingly, this tendency is observed mainly at the beginning of the task, that is, right after interacting with the black experimenter. Indeed, the same analysis conducted on the last two blocks of trials confirmed that the tendency to categorize black trustees was stronger at the end of the task, *F*(1,37) = 6.53, *p* = 0.02, $\eta_{p}^{2}$ = 0.15, when participants were no longer cooperating in a trial/error dynamic but rather according to what they had learned in the previous blocks of trials. The possibility that black trustees received greater attention is verified in Studies 2 and 3.

Overall, we could not provide empirical support to the results reported by Cañadas et al. (2015), who found the opposite pattern of data, i.e., categorization for the ingroup. In both Studies 1a and 1b, the ingroup-outgroup distinction was clearer than in Cañadas et al.’s (2015) report since it was manipulated according to only one social dimension. Therefore, our experimental design was likely more adequate to prove that intergroup context determines learning strategies.

Finally, in the first block of the second phase, in which new faces were presented at a 50% reciprocation rate, we observed that participants equally cooperated with new individuals from the social categories manipulated in the learning phase. The fact that we found no effect of the first cooperator factor (i.e., the manipulation of which group started reciprocating in a high proportion in the first phase of the experiment) confirms that participants did not learn to categorize, that is, they did not apply the cooperation biases introduced in the first phase to new individuals. However, they did learn something as the ingroup (Study 1a) or outgroup (Study 1b) favoritism found in the very first block of the experiment disappeared at the beginning of the second phase. Participants’ individual-based learning in Blocks 2–5 of the first phase transferred to new individuals in Block 1 of the second phase, in such a way that social categories became less significant as a criterion for decision-making. As a consequence, participants started individuating new trustees (i.e., Block 1 of the second phase) from the first interaction with them, in contrast with the initial block of trials.

In summary, Study 1 did not replicate the results reported by Cañadas et al. (2015) and rather indicated that when making decision about strangers, ingroup members are individuated whereas outgroup members are somehow categorized. Nonetheless, a possible experimenter effect causing the unexpected outgroup favoritism found in Study 1b still needs to be clarified. Study 2 aimed to test the experimenter effect and its possible consequences on participants’ perception of black trustees.

## Study 2

Study 2 investigated a possible effect of experimenter’s ethnicity which may have affected our data in Study 1b. Thus, we replicated the procedure of Study 1b manipulating between groups the ethnicity of the experimenter (White vs. Black). We expected participants to show in the first block of the first phase an ingroup favoritism with a white experimenter, and an outgroup favoritism with a black experimenter, as in Study 1b. Moreover, in line with Cañadas et al. (2015), we aimed at investigating whether the repeated interactions with the trustees in the trust game could affect the way they were perceived by participants in terms of trustworthiness and basic dimensions of social perception. For this reason, new measures of impression about trustees were included. Particularly, we expected trustees who individually reciprocated to be perceived as more trustworthy, and generally more positively than trustees who did not reciprocate. We also expected these data to echo the pattern from the trust game task in that the discrimination between high and low reciprocating trustees should be better for ingroup than for outgroup members. Finally, in line with our hypothesis that the presence of a black experimenter increased social regulation, we expected black trustees to be particularly attended, i.e., individuated, in the presence of a black experimenter, but not with a white one. The hypotheses, methods and analyses of this experiment were registered before data collection on Open Science Framework^5^.

### Method

#### Participants

##### Sample selection

Undergraduates from the local university were invited to participate in the experiment. Outside the classrooms, a paper–pencil list was handed over to several professors for students to sign up. This way, we ensured that participants had no contact with the experimenters before coming to the lab. For ethical reasons, men and foreign students were allowed to participate in the experiment but were excluded from the analyses in order to verify our hypotheses and to exclude the possibility of cultural or gender biases.

##### Sample size

On the basis of the analyses of Study 1b, we calculated an estimation of the adequate sample size with G*Power program v. 3.1.9.2 (Faul et al., 2007). In order to replicate the critical interaction corresponding to a better learning for ingroup vs. outgroup members, with an α-value of 0.05 and an estimated power of 0.90. The estimated effect size *f(V)* = 0.42 corresponded to the effect size found when the interaction reached significance, that is, when we included only the two last blocks of trials in the analysis (see the “Discussion” section of Study 1). We found that a sample of 39 participants per experimental group *(N* = 78) would be sufficient to replicate our data. In the end, 107 undergraduates (29 men/foreigners were excluded in line with our criterion of exclusion) (mean age: 19.23 years, range: 18–34 years) participated in exchange for financial compensation according to their accuracy in the task (5.81€ on average).

#### Apparatus and Stimuli

Apparatus and stimuli were identical to the ones used in Study 1b.

#### Procedure

The general procedure was identical to Study 1b, except that we introduced a post-interaction evaluation of participants’ impression about trustees. After performing the trust game, participants were asked to evaluate each of the 16 trustees on different social dimensions, as described in the next section.

Participants were distributed in the two experimental conditions (Experimenter: black vs. white) in the following way: Experimenters A (white woman) and B (black woman) ran the experiment on Tuesdays and Thursdays, and Mondays and Wednesdays, respectively, until reaching the sample size estimated as adequate. Experimenters provided participants with identical instructions, which they received in the same lab.

#### Measures

After performing the trust game, participants were asked to evaluate each one of the 16 trustees they had played with on a scale ranging from 1 “not at all” to 7 “totally” on the following dimensions: attractiveness, trustworthiness, competence, threat, and warmth, in line with Cañadas et al. (2015). In order to get a complementary measure of subjective categorization, participants were also asked about their subjective perception of similarity of each member with the rest of their group on a scale from -3 “very distinctive” to +3 “very indistinctive,” and the frequency with which they have been presented with each player in comparison with the others (1 “less,” 2 “the same,” 3 “more”). Moreover, participants were asked to indicate the perceived frequency of presentation and cooperation rate of each group in percentage.

Finally, apart from their impression about the trustees, participants were asked about their own general perception of intragroup similarity on a four-items scale ranging from 1 “not at all” to 7 “totally” with two items of low similarity (“It is easy to differentiate between black/white people,” “Most of black/white people are different”), and two items of high similarity (“It is hard to differentiate between black/white people,” “Most of black/white people are alike”). Two questionnaires of explicit prejudice toward blacks and women were included at the end of the experiment, but do not provide valuable information since participants showed extreme rates, likely for social desirability effects. Therefore, these data are not analyzed and not included in further experiments.

### Results

In line with Study 1, one participant was excluded from the analyses for having mean RT shorter than 200 ms in more than 50% of the trials, leaving in 77 participants for the analysis. Trials with RTs shorter than 200 ms (7%) were also excluded. As in Studies 1a and 1b, we first analyzed the cooperation rates in Blocks 1 of each phase in which participants were exposed to unknown faces, and then the cooperation rates resulting from the repeated interactions with them across Blocks 2–5 of both phases.

#### Cooperation Rates Prior to Learning: Block 1 of Each Phase

Cooperation rates in Block 1 of the first phase were introduced in a mixed-design ANOVA with trustee group (ingroup vs. outgroup) as a within-participants factor and experimenter (black vs. white) as a between-group variable. As in Study 1, we observed a main effect of trustee group indicating that participants cooperated more with black (*M* = 0.71, *SD* = 0.16, CI: 0.67–0.75) than with white trustees (*M* = 0.62, *SD* = 0.18, CI: 0.57–0.66), *F*(1,75) = 14.14, *p* < 0.001, $\eta_{p}^{2}$ = 0.16. This effect was not moderated by the ethnicity of the experimenter, *F* < 1, *p* > 0.50.

Furthermore, we aimed at verifying whether participants used their prior experience with trustees in order to make decision about new partners from the same category in Block 1 of the second phase. Thus, we conducted a 2 (trustee group: ingroup vs. outgroup) × 2 (first cooperator: ingroup vs. outgroup) × 2 (experimenter: black vs. white) mixed-design ANOVA. As in Study 1, we found no significant effect, all *F*s < 1.20, *p*s > 0.28.

#### Cooperation as a Result of Learning: Blocks 2–5 of Both Phases

A mixed-design ANOVA on cooperation rate was conducted with trustee group (ingroup vs. outgroup), group reciprocation (high vs. low), individual consistency (consistent vs. inconsistent) and blocks (2–5) as within-participants factors, and experimenter (black vs. white) as a between-participants variable. We found a significant Group Reciprocation × Individual Consistency × Blocks interaction, *F*(3,225) = 27.38, *p* < 0.001, $\eta_{p}^{2}$ = 0.27, indicating that, as in Study 1, participants used a strategy of individuation across blocks relying not only on trustees’ group reciprocation trend, but also on their individual consistency.

**FIGURE 3:**
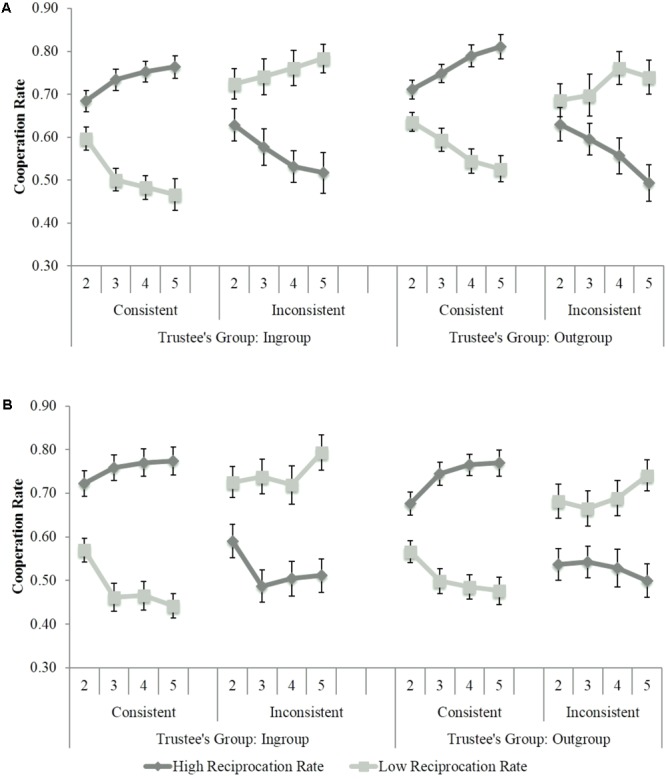
Participants’ cooperation rates in Study 2 as a function of trustees’ group, individual consistency and blocks of trials. **(A)** White Experimenter Condition. **(B)** Black Experimenter Condition.

We also observed a Trustee Group × Group Reciprocation × Individual Consistency interaction, *F*(1,75) = 3.70, *p* = 0.058, $\eta_{p}^{2}$ = 0.05. Although the Group Reciprocation × Individual Consistency interaction was significant for both ingroup, *F*(1,75) = 108.52, *p* < 0.001, $\eta_{p}^{2}$ = 0.59, and outgroup trustees, *F*(1,75) = 85.15, *p* < 0.001, $\eta_{p}^{2}$ = 0.53, the strategy of individuation was clearer for the former, as shown in **Figure 3**, and observed in Study 1.

#### Impression About Trustees

In order to analyze participants’ perception of trustees at the group level, scores on the perceived intragroup similarity, perceived frequency of presentation and perceived cooperation rates of each group (i.e., ingroup vs. outgroup) were introduced in different 2 (ethnicity: blacks vs. whites) × 2 (experimenter: black vs. white) mixed-design ANOVAs. We observed that participants perceived black trustees as more similar to each other than white individuals. Interestingly, they also perceived that black partners were presented more often and were more cooperative than white trustees, as shown in **Table 1**.

**Table 1:** Impression about trustees in Studies 2 and 3.

	Ingroup	Outgroup	*F*	*p*
**Study 2**				
Easy to discriminate	5.43 (1.41)	5.01 (1.48)	8.25	0.01**
Hard to discriminate	1.89 (0.80)	2.34 (1.18)	14.79	<0.001***
%Cooperation	42.69 (13.72)	56.70 (15.37)	24.36	<0.001***
%Presentation	46.70 (11.61)	52.19 (12.43)	5.32	0.02*
**Study 3**				
Easy to discriminate	5.42 (1.39)	4.92 (1.70)	5.3	0.025*
Hard to discriminate	2.08 (1.14)	2.23 (1.34)	0.8	0.38
% Cooperation	47.54 (14.91)	57.98 (14.66)	9.51	0.003**
% Presentation	47.67 (11.23)	56.93 (10.76)	12.12	0.001**

*Means and (standard deviations) of participants’ rates in the post-interaction evaluation of the social groups with whom they had interacted. *F*s and *p*s values correspond to the main effect of trustees’ social group (ingroup vs. outgroup) on participants’ rates in each scale.*

Then, we examined participants’ perception of trustees at the individual level. In order to verify whether the five dimensions measured (trustworthiness, attractiveness, competence, warmth, and perception of threat) could be classified into components, we conducted several principal components analyses (PCA) with an oblimin rotation. Each PCA was conducted in one of the condition resulting of the combination of our three within-subjects variables (trustee group, group cooperation and consistency), thus resulting in eight PCA. All Bartlett’s test of sphericity were significant [*p* < 0.001, smaller χ^2^(10) = 49.11], and Kaiser–Meyer–Olkin rates were high (smaller KMO = 0.55), thus it was acceptable to proceed with the analyses. In most of the PCA (five out of eight), the first component included the items trustworthiness, attractiveness, competence, and warmth while the fifth item measuring perception of threat was left in a second component. A reliability analysis revealed that Cronbach’s alphas were acceptable for the four items of the first component across the eight conditions (range: 0.59–0.81). Therefore, we averaged participants’ ratings of trustworthiness, attractiveness, competence and warmth to form the first component named impression, and separately analyzed their ratings at the scale measuring perception of threat.

Different within-subjects ANOVAs were conducted to examine participants’ impression about trustees (Component 1) as well as the perception of threat (Component 2). A significant Group Reciprocation × Consistency interaction was found on impression, *F*(1,75) = 19.75, *p* < 0.001, $\eta_{p}^{2}$ = 0.21, indicating that participants had a more positive impression about trustees who individually highly cooperated (*M* = 4.03, *SD* = 1.01), than trustees who did not (*M* = 3.62, *SD* = 1.13). Moreover, the Trustee Group × Group Reciprocation × Consistency interaction was marginal, *F*(1,75) = 3.16, *p* = 0.08, $\eta_{p}^{2}$ = 0.04, echoing the results found in the trust game: despite participants learnt to individuate both outgroup, *F*(1,75) = 9.59, *p* = 0.003, $\eta_{p}^{2}$ = 0.11, and ingroup, *F*(1,75) = 22.06, *p* < 0.001, $\eta_{p}^{2}$ = 0.23, the effect was larger for the latter. Finally, a main effect of Experimenter, *F*(1,75) = 5.56, *p* = 0.021, $\eta_{p}^{2}$ = 0.07, showed that overall participants had a more positive impression about trustees when the experimenter was black (*M* = 4.03, *SD* = 1.03) than when she was white (*M* = 3.62, *SD* = 1.10).

On the second component, perception of threat, a significant Group Reciprocation × Consistency interaction was found, *F*(1,75) = 15.99, *p* < 0.001, $\eta_{p}^{2}$ = 0.18, revealing that in line with previous data, participants perceived trustees who individually cooperated more as less threatening (*M* = 2.33, *SD* = 1.30) than trustees who tended not to cooperate (*M* = 2.76, *SD* = 1.57).

### Discussion

Study 2 replicated Study 1b while examining the effect of the experimenter’s ethnicity on participants’ decision whether to trust black and white unknown game partners.

Prior to experience, participants cooperated more with black than with white partners, independently of the experimenter’s ethnicity. Therefore, the presence of a black experimenter is not sufficient to explain why participants were more cooperative with black individuals. Despite it has been repeatedly argued that ingroup members are perceived more positively than outgroup members (e.g., Brewer, 1981, 2007), it is not the first time that an outgroup favoritism is reported in trust decisions. Tortosa et al. (2013a) conducted a series of two trust game experiments in which participants played with black and white trustees. In the first one, participants equally cooperated with black and white partners. However, in the second experiment, the authors found a tendency to cooperate more with black individuals than with white ones. Interestingly, they also observed an implicit negative bias toward black people in a Blacks/Whites Implicit Association Task (Greenwald et al., 1998) leading the authors to interpret the data from the trust game task as an attempt from participants not to show their prejudice-related biases in an explicit task, by appearing more cooperative with black individuals. A similar conclusion may be drawn from our data. Despite the apparent positive attitude toward black partners in the first block of the experiment, participants learned more efficiently the cooperative trends of white trustees while their decisions about black partners were somehow influenced by the group information. Therefore, it is possible that the higher cooperation rates with black trustees reflected an active effort from our participants to be perceived as egalitarian people (Dunton and Fazio, 1997; Maddux et al., 2005; Kawakami et al., 2009; Nosek et al., 2011).

We also replicated and confirmed the data from Study 1b corresponding to a pattern of individuation stronger for ingroup than for outgroup members. Particularly, we found that participants’ decision (not) to cooperate was made according to trustees’ individual reciprocation trend when they were white independently of the group reciprocation trend. However, participants identified less efficiently black trustees’ reciprocation trends when they were inconsistent with the other members of their group. Interestingly, these results were not moderated by the experimenter’s ethnicity. Therefore, the discrepancy between our pattern of results and the data reported by Cañadas et al. (2015) is unlikely due to the experimenter’s ethnicity.

In fact, our results are rather consistent with the prior literature stating that outgroup members are mostly perceived according to their group membership (Billig and Tajfel, 1973; Haslam et al., 1996). This interpretation is supported by the results from the post-interaction impression about trustees, since participants indicated they perceived black people as more similar to each other than white people. Moreover, the general perception of black partners within the game was somehow biased in that participants also perceived that black partners were presented more often and were more cooperative than white people. This was not the case since all trustees were equally presented and each group equally reciprocated. According to Fiske (1980), unusual or extreme stimuli are more salient. Given the scarcity of black people in the social context where the study took place, they may have received more attention in the task such that participants overestimated the frequency of presentation or reciprocation of black trustees. Still, this general striking effect did not encourage individuating processes.

Finally, the individuation strategy observed in the trust game was confirmed in the post-interaction evaluation of trustees. Participants’ perception of trustees was affected by their individual reciprocation trend in the two components evaluated (positive impression and perception of threat), although this individuation strategy tended to be greater the ingroup, as reflected in the marginal three-way interaction, in line with the data observed in the trust game.

In summary, Study 2 allowed to replicate the data from Study 1b and to rule out the presence of an experimenter effect. However, it remains unclear whether the discrepancy between our results and the data reported by Cañadas et al. (2015) is explained by the overlap between gender and ethnicity in their study. We address this issue in Study 3.

## Study 3

Across Studies 1 and 2, we found that, despite participants mostly individuated their game partners, this pattern was poorer when it came to outgroup members. These results are consistent with our hypothesis and the literature on social cognition (Tajfel and Wilkes, 1963) but inconsistent with the data reported by Cañadas et al. (2015). Thus, we decided to verify whether the confusion between gender and ethnicity among trustees was responsible for the pattern of data reported by Cañadas et al. (2015). We conducted an exact replication of their experiment in which men and women were presented in each ethnic group, while at the same time controlling for the ethnicity of the experimenter as done in Study 2. If having a white experimenter is crucial for replicating the pattern of data observed by Cañadas et al. (2015), we should replicate it with a white experimenter whereas we should obtain the pattern of data observed in Studies 1 and 2 with a black experimenter. The hypotheses and method of this experiment were registered before data collection on Open Science Framework^6^.

### Method

#### Participants

##### Sample selection

The sample selection was identical as in Study 2, except that data from men were included in the analyses and only data from foreign students were excluded. This change in the sample selection was introduced since in this study, only ethnicity determined trustee’s group membership with respect to participants. In other words, gender was no longer relevant for discriminating between ingroup and outgroup members among trustees, neither was among participants.

##### Sample size

We calculated an estimation of the sample size necessary in order to replicate the data reported by Cañadas et al. (2015) using the same parameters as described by the authors [α-value of 0.003, estimated power of 0.90 and estimated effect size *f(V)* = 0.65] with G*Power program v. 3.1.9.2 (Faul et al., 2007). We found that a minimum of 29 participants per experimental group (*N* = 58) would be sufficient to replicate the significant three-way interaction corresponding to a better learning for outgroup than for ingroup members. Finally, 67 undergraduates (62 excluding foreigners) (11 males, mean age: 20.21 years, range: 18–27 years) voluntarily participated in exchange for financial compensation according to their performance in the task (5.61€ on average).

#### Apparatus and Stimuli

The same photographs of eight white and eight black trustees (half men in each ethnic group) used by Cañadas et al. (2015) were used in this experiment.

As for Study 2, a white and a black experimenter were provided with the same instructions to the participants, in the same lab, and alternated weekly until reaching the right number of participants in each group. In order to reduce the duration of the task and given that the dimensions measured in Study 2 provided similar information, the dimensions evaluated in this study were reduced to trustworthiness and attractiveness, as in Cañadas et al. (2015).

### Results

Four participants were excluded from the analyses for having a mean RT faster than 200 ms in more than half of the trials, leaving in 58 participants for the analyses. Trials with RTs shorter than 200 ms (11%) were also excluded from the analyses. Cooperation rates in Block 1 of each phase are presented, followed by the cooperation rates resulting from the repeated interactions with trustees across Blocks 2–5 of both phases. All the analyses were identical to Study 2.

#### Cooperation Rates Prior to Learning: Block 1 of Each Phase

A mixed design ANOVA with trustee group as a within-subjects variable and experimenter as a between-group factor revealed a main effect of trustee group indicating that participants cooperated more with outgroup (*M* = 0.66, *SD* = 0.23, CI: 0.60–0.72) than with ingroup members (*M* = 0.60, *SD* = 0.22, CI: 0.54–0.65), *F*(1,56) = 4.61, *p* = 0.04, $\eta_{p}^{2}$ = 0.08, independently of the experimenter, *F*(1,56) = 0.08, *p* = 0.78, in Block 1 of the first phase. A 2 (trustee group: ingroup vs. outgroup) × 2 (first cooperator: blacks vs. whites) × 2 (experimenter: black vs. white) mixed-design ANOVA on cooperation rates in Block 1 of the second phase showed no significant effect, *Fs* < 1.33, *p*s > 0.25.

#### Cooperation Rates as a Result of Learning: Block 2–5 of Both Phases

**FIGURE 4:**
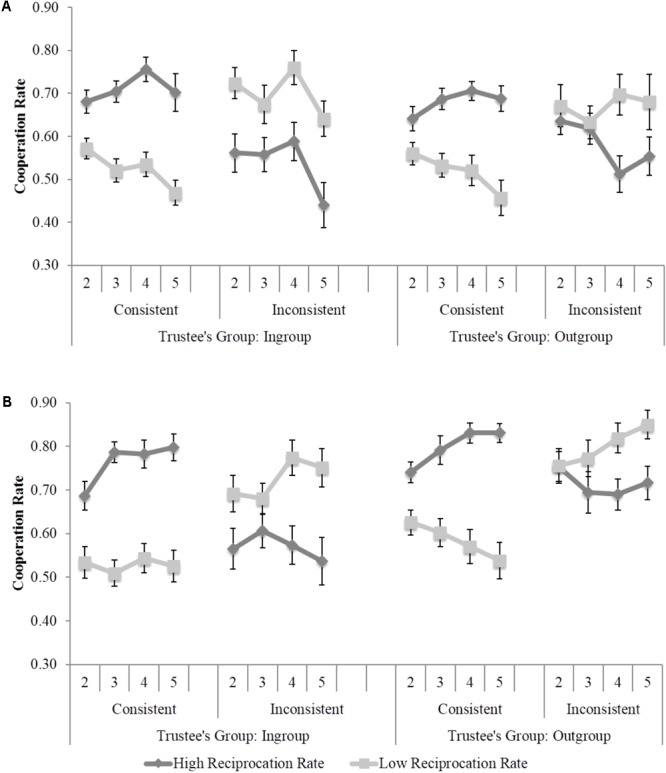
Participants’ cooperation rates in Study 3 as a function of trustees’ group, individual consistency and blocks of trials. **(A)** White Experimenter Condition. **(B)** Black Experimenter Condition.

Cooperation rates in Blocks 2–5 of both phases were introduced in a mixed-design ANOVA with trustee group (ingroup vs. outgroup), group reciprocation (high vs. low), individual consistency (consistent vs. inconsistent) and blocks (2–5) as within-participants factors, and experimenter (black vs. white) as a between-participants variable. Once again, we found a Group Reciprocation × Individual Consistency × Blocks interaction, *F*(3,165) = 15.04, *p* < 0.001, $\eta_{p}^{2}$ = 0.22, showing that individuation was preferred over categorization along the blocks of trials. Moreover, the Trustee Group × Group Reciprocation × Individual Consistency interaction was again replicated in this experiment, *F*(1,55) = 4.95, *p* = 0.03, $\eta_{p}^{2}$ = 0.08, indicating that despite participants individuated both ingroup, *F*(1,55) = 43.27, *p* < 0.001, $\eta_{p}^{2}$ = 0.44, and outgroup, *F*(1,55) = 37.78, *p* < 0.001, $\eta_{p}^{2}$ = 0.41, the pattern of individuation was clearer for ingroup members as shown in **Figure 4**. This effect was not modulated by the experimenter variable, *F*(1,55) < 0.01, *p* = 0.98, $\eta_{p}^{2}$ < 01.

#### Impression About Trustees

The same analyses as in Study 2 revealed that black trustees were perceived as more similar to each other than white trustees. Participants also perceived that black game partners were presented more frequently along the task, and were more cooperative than white trustees, as seen in **Table 1**.

As in Study 2, participants’ rates to the scales evaluating trustworthiness and attractiveness were averaged in a unique component evaluating positive impression. A mixed-design ANOVA revealed that participants evaluated the trustees according to their individual reciprocation trend, as reflected in the Group Reciprocation × Consistency interaction *F*(1,56) = 28.54, *p* < 0.001, $\eta_{p}^{2}$ = 0.34. Trustees who individually cooperated at a high level were perceived more positively (*M* = 3.69, *SD* = 1.43) than trustees who individually tended not to cooperate (*M* = 3.03, *SD* = 1.06). Moreover, this interaction was modulated by trustee group, *F*(1,56) = 7.83, *p* = 0.007, $\eta_{p}^{2}$ = 0.12. Despite participants evaluated more positively trustees who individually reciprocated more for both ingroup, *F*(1,56) = 36.71, *p* < 0.001, $\eta_{p}^{2}$ = 0.40, and outgroup, *F*(1,56) = 11.58, *p* = 0.001, $\eta_{p}^{2}$ = 0.17, this individuation was clearer for the ingroup than for the outgroup. We also found a Experimenter × Consistency interaction, *F*(1,56) = 7.61, *p* = 0.008, $\eta_{p}^{2}$ = 0.12, showing that the general more positive impression observed with a black experimenter than with a white experimenter observed in Study 2 was replicated here only for inconsistent, *F*(1,56) = 3.87, *p* = 0.054, $\eta_{p}^{2}$ = 0.07, but not for consistent trustees, *F*(1,56) = 0.07, *p* = 0.80, $\eta_{p}^{2}$ < 0.01.

### Discussion

In Study 3, we conducted an exact replication of the experiment reported by Cañadas et al. (2015), while manipulating experimenters’ ethnicity as in Study 2. In line with the previous experiments, we found that participants cooperated more with black than with white trustees before learning their particular reciprocation tendency. Interestingly, they later showed a tendency to categorize black individuals as they were less efficient at learning about inconsistent black game partners. The positive impression toward black trustees at the beginning of the experiment does not seem to transfer into a better learning of their particular reciprocation rates. It is possible that in the first block, participants were particularly motivated to be egalitarian and actively showed a high readiness to cooperate with black trustees because of the social norms against racial prejudice. This would be consistent with the evidence of discrepancies between explicit intention to be egalitarian and the implicit negative bias against black people (Kawakami et al., 2009; Nosek et al., 2011). In our studies, explicit intention to be egalitarian may be observed in high cooperation rates with black trustees while negative implicit biases against black people may be reflected in a subsequent category-based learning about them. The fact that we used an infrequent outgroup in the social context where the study took place may have increased this tendency. In future research, it would be convenient to control for the frequency of contacts with outgroup members. Otherwise, the inclusion of an implicit measure of prejudice toward black people may help to disambiguate whether there is a negative implicit attitude toward black people subjacent to the initial positive attitude toward black people observed here.

Further, we replicated the pattern of learning observed in our previous studies. While participants clearly individuated ingroup members, they rather seemed to categorize outgroup members to some extent. These results are consistent with previous literature arguing that outgroup members are perceived in a more categorical way than ingroup members (e.g., Tajfel and Wilkes, 1963; Park and Rothbart, 1982; Haslam et al., 1996). However, we could not provide an empirical replication of the results reported by Cañadas et al. (2015). Despite using the same experimental procedure and materials as Cañadas et al. (2015), and controlling for other variables such as the experimenter’s ethnicity or the instructions across the experiments, we did not replicate the pattern of categorization for ingroup and individuation for outgroup members described in their report. The fact that the pattern of data presented in the present research is consistent with the literature and replicated across the three experiments leads us to believe that it is reliable, while the reasons why Cañadas et al. (2015) found a different pattern of results remain unclear.

**FIGURE 5:**
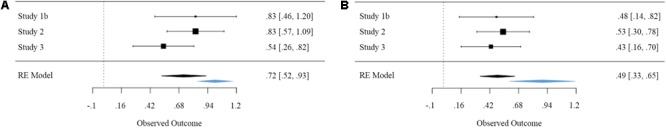
Confidence interval of the effect sizes in each study and confidence intervals of the meta-analytic average. Negative values would correspond to category-based learning and positive values to individual-based learning. Blue areas represent the effect size observed with consistent individuals, 0.97 and 0.89, respectively, for ingroup and outgroup trustees. **(A)** Ingroup Inconsistent Condition. **(B)** Outgroup Inconsistent Condition.

In order to shed light on these discrepancies, we meta-analyzed Studies 1b, 2, and 3 and the original study reported by Cañadas et al. (2015) using random-effects model and the mean effect size of the *t*-test comparisons of cooperation rates with low vs. high reciprocating trustees, separately for ingroup and outgroup inconsistent members. The pattern of categorization for ingroup members observed by Cañadas et al. (2015) was not supported. Indeed, across the four studies, the strategy of individuation was significant for both ingroup, *Z* = 1.95, *p* = 0.050, and outgroup, *Z* = 6.54, *p* ≤ 0.001. However, in the ‘ingroup members’ condition, we observed a very high heterogeneity, *I*^2^ = 88.89%, τ^2^ = 0.26, with a significant Cochran’s *Q* = 23.58, *p* < 0.001, which was drastically reduced when removing Cañadas et al.’s (2015) study, *I*^2^ = 29.07%, τ^2^ < 0.01, *Q* = 2.68, *p* = 0.26. Influential case diagnostic confirmed that Cañadas et al.’s (2015) study had a strong influence on the results reflected in large DFBETAS (DFBETAS = -2.48). New meta-analyses removing Cañadas et al.’s (2015) study confirmed the consistency of the results across the three experiments of the current research with a significant individual learning for both ingroup and outgroup members, larger for ingroup members, as seen in **Figure 5**.

A possible relevant difference between Cañadas et al.’s (2015) study and our Study 3 is the sample. Despite we could control that all participants were born and grown in the same cultural context, it is possible that a group of participants (i.e., the sample used by Cañadas et al., 2015) was by chance more motivated to individuate outgroup members. Indeed, participants indubitably bring to the lab their real life goals, which may be reflected in their performance in an experimental task if it is related enough. With a sample of undergraduates, several circumstances may have increased participants’ will to perceive outgroup members in a more individuated manner such as the area of study that may be more or less related to social concerns, or a particular class about social categorization, prejudice or discrimination. Given the impossibility of predicting the particular motivational context of each one of the participants, future studies should include measures of attitudes toward the outgroup, as well as motivation for controlling prejudices, in order to assess individual differences. In addition, systematic replication is a useful strategy to control for broader contextual variables.

An interesting result is that in Study 3, as in the previous one, participants perceived that black trustees were presented more often, were more cooperative and were more similar to each other. The scarcity of contact between our participants and black people may account for these results. Finally, in line with our hypothesis and the data from the trust game, we found that trustees who individually reciprocated were perceived more positively than trustees who individually did not, reflecting an individuated impression about trustees. This effect was clearer for white than for black trustees, thus showing that the individuation processes observed in the trust game seem to transfer to the subsequent explicit evaluation of trustees in the exact same way.

In summary, Study 3 replicated the data found in Studies 1b and 2 indicating that in a trust decision-making, ingroup members are highly individuated whereas the decision to trust outgroup members is also guided by behavior of the group they belong to. These results appeared to be independent of the experimenter’s ethnicity or of the presence of male and female trustees within each group.

## General Discussion

Across three experiments, we investigated whether participants used individuation or categorization processes to interact with strangers in a trust game paradigm. Our results generally support the hypothesis that when deciding whether or not to trust individuals with whom they had no prior experience, participants quickly identified and used social categories to make their decisions.

The first important result of the current set of studies comes from the data from the first block of the experiments, that is, when participants were presented with unknown people. Gender and ethnicity manipulations resulted in different patterns of responses. When gender was manipulated, an ingroup favoritism was found, that is, participants trusted to a greater extent the people of the same group membership as themselves (Study 1a). However, when ethnicity was manipulated, participants showed an unexpected outgroup favoritism, in that they trusted more outgroup than ingroup members (Studies 1b, 2, and 3).

The results from the manipulation of gender support the hypothesis that ingroup is generally perceived more positively than outgroup (e.g., Brewer, 2007). However, the outgroup favoritism for black trustees was rather unexpected. In this sense, it is interesting that after performing the trust game, participants inaccurately perceived black trustees as being presented more often and being more cooperative than white trustees. Therefore, it seems that participants’ perception of black trustees was generally biased, possibly because the population of black people is very scarce in the context where the experiment took place and thus more salient (Fiske, 1980). For this reason, participants may have been particularly motivated to be perceived as equalitarian people (Plant and Devine, 1998), and in consequence cooperated to a great extent with black trustees.

A second key result is that across repeated interactions, participants learnt about trustees and their decisions were influenced by individuation and categorization strategies during learning. Individuation was the general trend with all trustees. However, learning about outgroup (men in Study 1a and Blacks in Studies 1b, 2, and 3) was also affected by categorical thinking. Outgroup members were somehow categorized, as participants could not learn whether or not to trust outgroup individuals deviating from the group reciprocation tendency, as efficiently as they learnt about ingroup members. This result is consistent with the predictions of the outgroup homogeneity effect which state that we have a more categorical perception of outgroup than ingroup members (Tajfel and Wilkes, 1963). The categorization of black individuals was confirmed in participant’s post-interaction impression about trustees in Studies 2 and 3 since, black individuals were perceived as more similar to each other than white individuals. Moreover, while white trustees who individually reciprocated were easily identified and consistently evaluated as more attractive than white trustees who did not, the evaluation of black trustees’ attractiveness was inconsistent across the studies and did not seem to depend reliably on their individual reciprocation tendency. Together, these data suggested an important effect of intergroup context in trust decision-making: white participants failed to fully individuate black partners (i.e., men and women) when they were presented together with white partners, in the same way that women failed to individuate male partners, when they were presented together with female partner.

There are a number of implications of these results for real life relationships. When categorical judgments affect trust decisions, the inferences made about strangers are necessarily less accurate and the perception of the outgroup is mistaken. In intergroup contexts, such inaccuracies may become particularly important since they prevent people from establishing reliable trust relationships. The inefficiency to learn about outgroup members may result in a more negative perception of the group, opening the path to intergroup conflicts and prejudice.

### Limitations and Further Directions

Despite shedding light on the processes underlying social learning in intergroup contexts, our studies also let some questions open. For instance, the reasons why we observed an unexpected outgroup favoritism across Studies 1b, 2, and 3 are still to clarify. Further research may address this issue by using a more frequent outgroup, measuring participants prejudice toward black people (see the experimental procedure used by Tortosa et al., 2013a), their motivation for controlling prejudice, or manipulating trustees’ and/or participants’ ethnicity. A complementary option is to use a general population sample who is less likely familiarized with social perception concerns compared to the undergraduate samples used in the present studies.

The need for maintaining a clear ingroup–outgroup distinction between trustees and participants led us to use a white female sample in Study 1, which limits the generalizability of the results to a broader population. When manipulating ethnicity, this limitation was addressed in Study 3 in which men and women participated in the experiment and showed similar results as in Studies 1b and 2. However, regarding our manipulation of gender, this issue should be addressed in future research by conducting the same experiment with a male sample of participants.

The present research did not replicate the data reported by Cañadas et al. (2015), although it is consistent with the previous literature on outgroup categorization. It is possible that Cañadas et al. (2015) pattern of results is spurious, or that our samples of participants differed from the sample used by Cañadas et al. (2015) on one or several dimensions such as the level of prejudice toward black people or the motivation for controlling prejudice. Controlling the aforementioned variables related to the sample would shed light on these potential explanations. Importantly, the discrepancies between different studies in the process of replication actually help to deepen our knowledge of a particular topic. What is often considered as a *failure* to replicate may rather be a step ahead in the understanding of the specific variables and circumstances that affect the results, an issue of the most importance which can hardly be addressed in a single study (see Open Science Collaboration, 2015, for a similar purpose).

In any case, the procedure developed by Cañadas et al. (2013) and extended in this paper is a remarkable contribution in the study of individuation and categorization processes. The inclusion of an inconsistent member within a social group is a key manipulation to understand to what extent the group knowledge may be generalized to all individuals in a stereotyped manner, independently of their particular behavior. It appeared to be a useful tool to investigate these processes in different social contexts (emotion: Cañadas et al., 2016; ethnicity: Cañadas et al., 2015; gender: Cañadas et al., 2013) and related to different DVs such as RT or cooperation rates, thus granting its external validity. Further research could reliably use this paradigm to examine the use of individual- or category-based strategies.

## Conclusion

In conclusion, our results broadly indicated that, in impression formation processes, social categories play a key role in our interactions at zero acquaintance, but also along repeated interactions when we learn about them. Social categorization may occur even when participants are highly motivated for individuating, and provided with the means to do so. The current results suggest that in cooperation settings, the information that categorizes people with whom we interact might sometimes bias our decisions and hinder our performance.

## Ethics Statement

This research was conducted according to the guidelines set forth by the University of Granada on the use of human participants in research. This research is part of a larger research project approved by the University of Granada ethical committee (175/CEIH/2017).

## Author Contributions

MT, EC, RR-B, and JL conceived and designed the studies. MT and EC programmed the experiments. MT performed the experiments. MT, RR-B, SdL, and JL analyzed and interpreted the data. MT wrote the manuscript that was critically revised by EC, SdL, RR-B, and JL.

## Conflict of Interest Statement

The authors declare that the research was conducted in the absence of any commercial or financial relationships that could be construed as a potential conflict of interest.

## Footnotes

1 After data collection, we realized there was a mistake in the gender manipulation condition. One photograph of a male trustee was repeated, such that participants played with three instead of four trustees. This condition was replicated with forty new participants. Here, we present the data of the corrected new experiment. As a consequence, the original between-group manipulation is therefore presented as two different experiments: Study 1a and Study 1b.

2 At the time this experiment was carried out, it was not usual in our lab to perform power analyses to estimate sample size. However, a sensitivity power analysis assuming an alpha criterion of 0.05 and a power criterion of 0.80 revealed that with our sample of 40 and 41 participants, respectively, in Studies 1a and 1b, the smallest effect size that could have been detected for the critical Trustee Group × Group Reciprocation × Individual Consistency interaction was 0.23.

3 The photographs were ceded by the Social Cognition Lab of York University.

4 Accurate trials were those in which participants’ decision matched trustees’ reciprocation or deception.

5 osf.io/6dqs7

6 osf.io/tz7c8
